# Unique form of catheter malconnection following intrathecal baclofen surgery for spinal cord injury: a case report

**DOI:** 10.1007/s00701-023-05718-z

**Published:** 2023-07-21

**Authors:** Raoul J. Koopmans, Carel G. M. Meskers, Vincent de Groot, K. Mariam Slot

**Affiliations:** 1 Department of Neurosurgery, Amsterdam UMC Location University of Amsterdam, Meibergdreef 9, Amsterdam, The Netherlands; 2 Department of Rehabilitation Medicine, Amsterdam UMC Location Vrije Universiteit Amsterdam, Boelelaan 1117, Amsterdam, The Netherlands; 3 Amsterdam Movement Sciences, Rehabilitation & Development, Amsterdam, The Netherlands

**Keywords:** Disconnection, Dysfunction, Malfunction, Intrathecal baclofen (ITB), Spinal cord injury (SCI), Case report

## Abstract

This case report concerns a patient suffering from traumatic spinal cord injury with severe spasticity treated with intrathecal baclofen therapy. After revision surgery for a confirmed catheter obstruction, progressive spasticity reappeared. Diagnostics demonstrated signs of catheter fracture or disconnection adjacent to the pump. During revision surgery, the silicone layer surrounding the sutureless pump connector was shown to be curled up, revealing the cause of dysfunction. As far as we know, this form of malconnection has not been reported before. Therefore, surgeons must be aware of this complication and additional inspection of the silicone connector prior to definite connection is advised.

## Introduction

Spinal cord injury (SCI) can lead to extensive limitations in function and quality of life. Spasticity is highly prevalent in SCI and can further increase these limitations. In addition, it can result in pain, contractures, pressure ulcers, and infections which further impairs functioning [7, 9]. Treatment of spasticity is usually first attempted through nonpharmacological treatment such as physiotherapy, after which oral antispasticity drugs such as baclofen are administered if spasticity is widespread [1, 9].

Oral baclofen crosses the blood–brain barrier ineffectively and subsequently has a low intrathecal bioavailability. As a result, oral dosages may need to be high and systemic side effects are common and dose limiting [14]. When oral therapy remains insufficient, intrathecal baclofen (ITB) should be considered next [8, 13]. ITB is administered by a surgically implanted subcutaneous pump with a catheter extending from the pump into the intrathecal space, hereby bypassing the blood–brain barrier resulting in a higher intrathecal bioavailability with a relatively small dosage, ensuing reduced spasticity without the troublesome systemic side effects [8, 9, 13].

Unfortunately, complications after ITB surgery (e.g., drug withdraw, drug overdose, hemorrhage, infection, cerebrospinal fluid (CSF) leakage, pump malfunction, and catheter-related complications) occur frequently and often require re-operation [5, 10]. The most common ITB system-related complications are catheter-related complications (CRC) which comprise kinking, occlusion, migration, fracture, and disconnection and occur in 4–20% of patients within one year after surgery [2, 5].

This paper presents a unique, subtle form of catheter malconnection resulting in the ineffectiveness of a SynchroMed II pump from Medtronic with corresponding Ascenda catheter, in a patient suffering from traumatic SCI-related spasticity. To the best of our knowledge, this complication has not been reported before.

## Case report

A 40-year-old male suffered from an incomplete traumatic SCI after a car accident in 2006 resulting in tetraplegia without motor function, but partial intact sensibility below the level of C6. Because of progressive spasticity and contractures resulting in pain and limitations in functioning, an ITB pump was surgically implanted in 2009 and electively revised in 2015 because of end of battery life.

In June 2021, the patient slowly developed intermittent, progressive painful spasticity with frequent spontaneous clonic reflexes in the lower extremities. After excluding other potential causes of progressive spasticity such as constipation or urinary tract infection, an intermittent malfunction of the ITB system was suspected, and an obstruction of the intrathecal part of the catheter was diagnosed through an Indium (In111 DTPA) Single-Photon Emission Computed Tomography (SPECT) study [11]. After diagnosis, oral antispasticity drugs were administered for a short period of time after which spasticity-related symptoms improved. In February 2022, spasticity-related symptoms returned, and the ITB pump including spinal catheter was electively revised in March 2022. Before closure of the wounds, the integrity of the newly implanted system was verified via side port procedure, whereby saline was flushed through the side port into the catheter without any signs of leakage or obstruction.

**Fig. 1:**
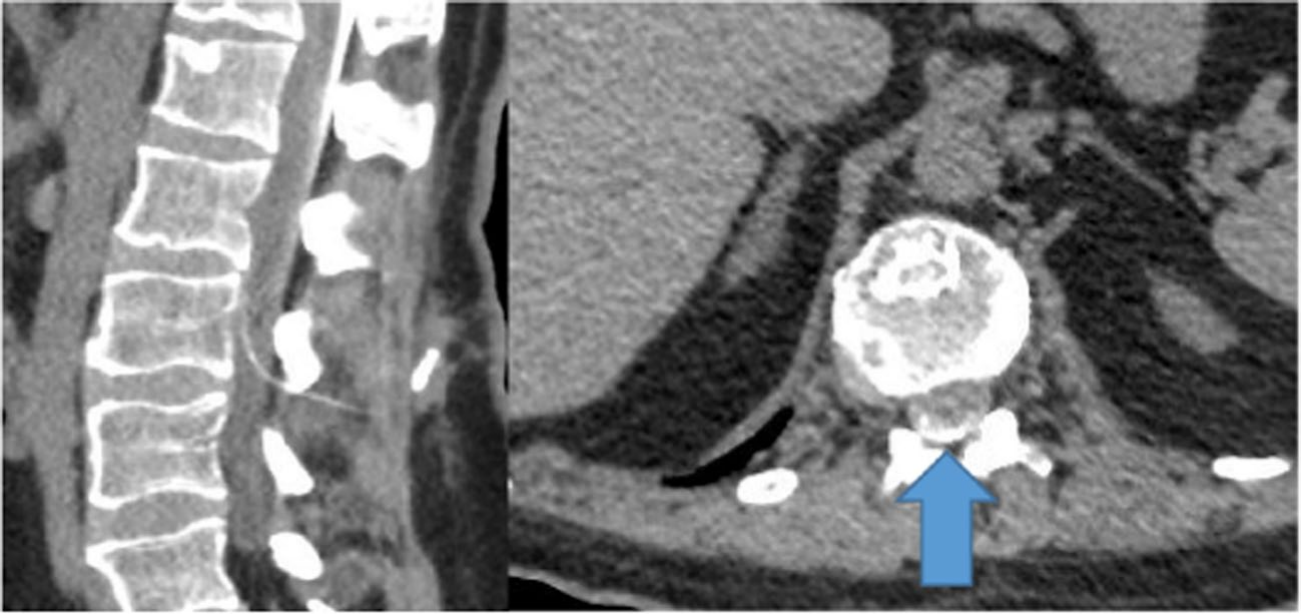
Intrathecal contrast after catheter access port contrast injection on CT (blue arrow)

**Fig. 2:**
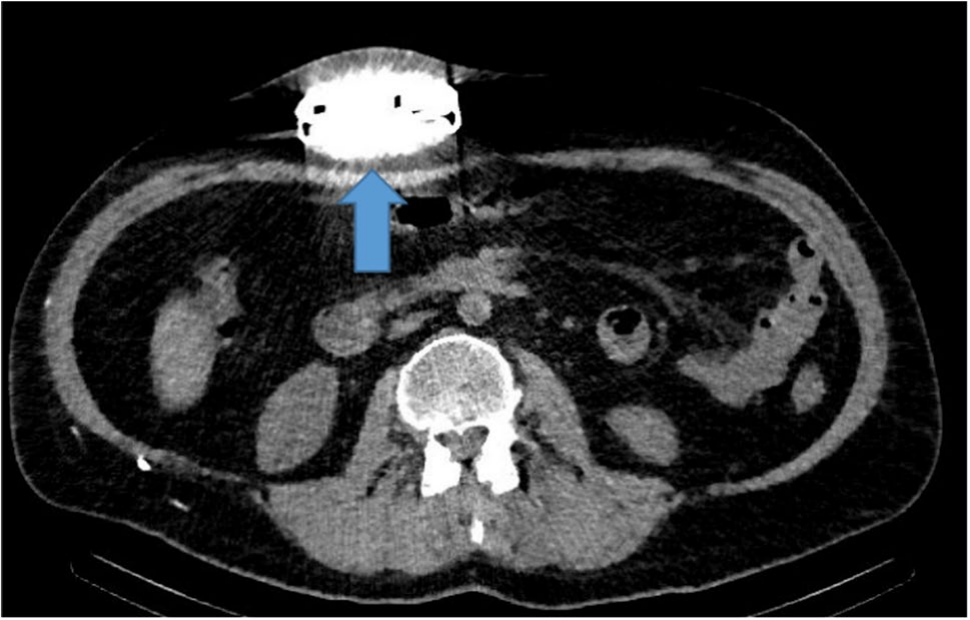
Edema (blue arrow) and suspected fluid collection on CT

Postoperative, there were no signs of complications within the first week. However, intermittent painful spasticity accompanied by headaches while seated appeared a week after surgery, highly suspected for recurrent dysfunction. Conventional radiography demonstrated no obvious disconnections, and CSF aspiration was feasible via side port procedure. Subsequently, CT was carried out, whereby contrast was injected into the ITB pump catheter via the catheter access port. This demonstrated catheter patency with the appearance of intrathecal contrast. No extravasation of contrast or visible fluid collections surrounding the catheter or pump that could indicate dysfunction or CSF leakage were observed (Fig. 1). Oral baclofen was given to reduce symptoms, but showed insufficient clinical improvement on spasticity after which a diagnostic intrathecal bolus of 50 mcg baclofen was administered through the pump, without any clinical improvement on spasticity. The next step was an Indium (In111 DTPA) SPECT study. This study demonstrated no intrathecal or intracatheter indium, accompanied by a suspected fluid collection around the pump. After aspiration of the fluid, laboratory tests confirmed the fluid to be CSF and baclofen (Fig. 2). Consequently, elective revision was planned.

**Fig. 3:**
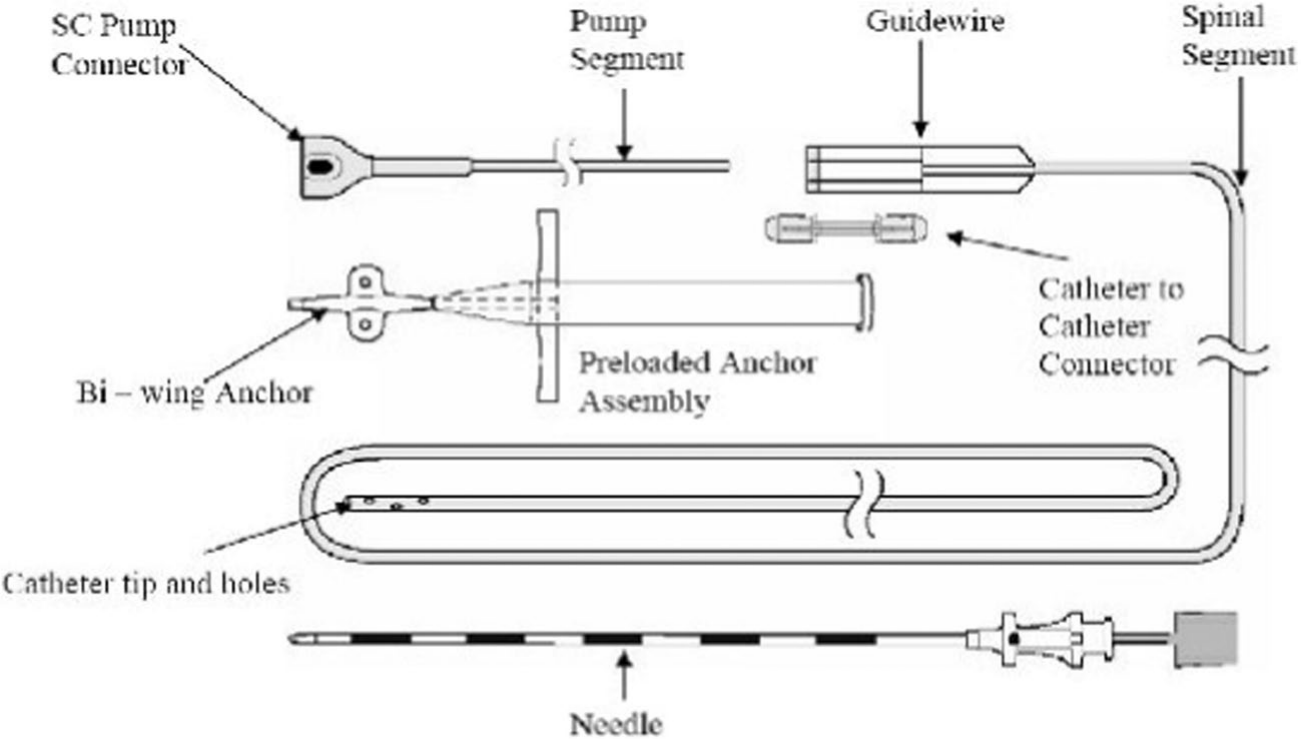
Medtronic Ascenda catheter system

**Fig. 4:**
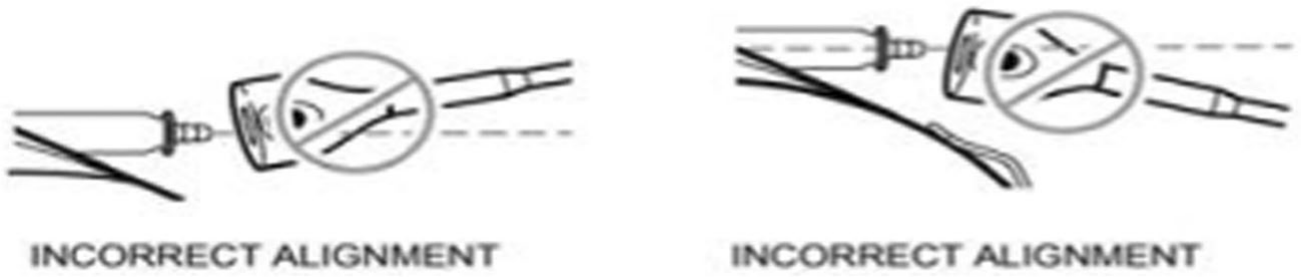
Incorrect alignment of the sutureless pump connector

**Fig. 5:**
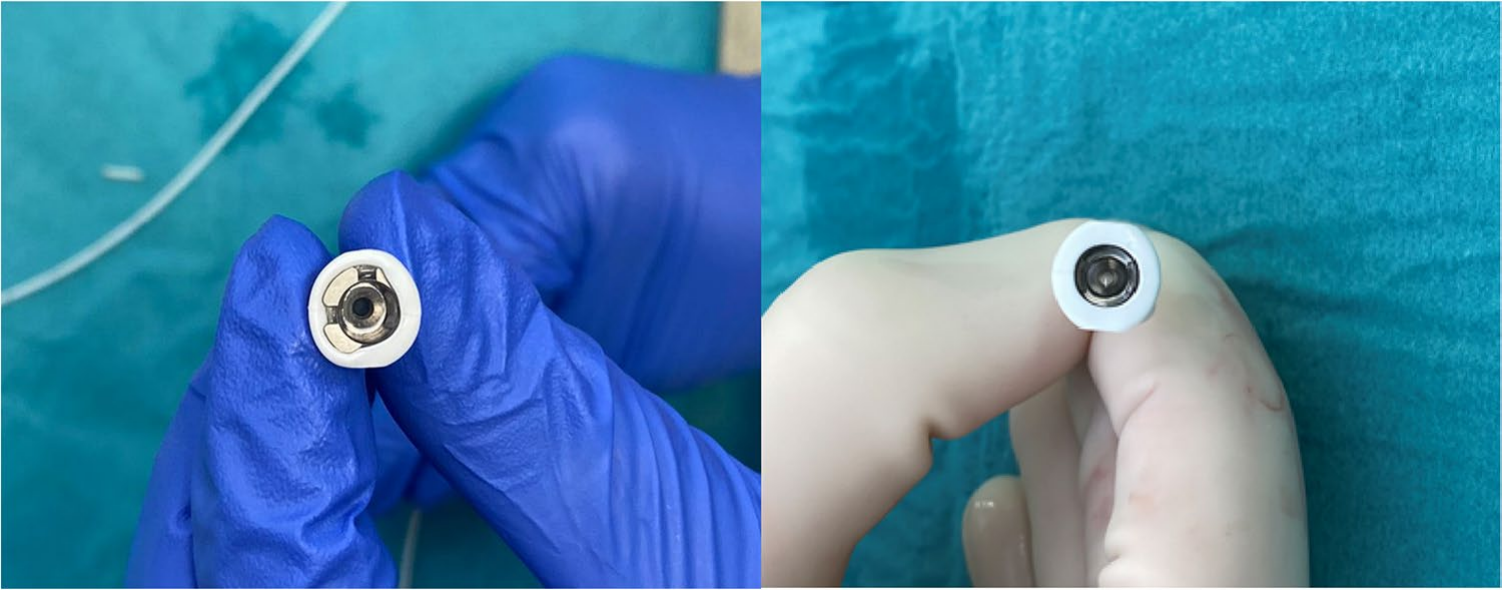
Curled up silicone layer surrounding the sutureless pump connector (left) and normal silicone layer surrounding the sutureless pump connector (right)

On opening of the abdominal subcutaneous pocket, CSF emerged. After mobilizing and removing the pump for inspection, no disconnection, tear, or active leakage was observed. However, after disconnection of the well-connected and aligned sutureless pump connector (Fig. 3 and 4), the silicone layer surrounding the sutureless pump connector, ensuring watertight connection between the pump and catheter, was shown to be curled up, illuminating the probable cause of dysfunction (Fig. 5). After verifying functionality of the spinal catheter, without signs of dysfunction, the part of the catheter connected to the pump was replaced; after which patency was confirmed through side port procedure and the pump was re-implanted subcutaneously. The postoperative period was uneventful, and the patient was discharged a day after surgery with improvement of spasticity symptoms. Two weeks after surgery, the patient was followed up in the outpatient clinic in accordance with standard postoperative care, without any signs of complications.

## Discussion

In this case, ITB system malfunction with CSF leakage was suspected because of recurrent spasticity and headaches after a period of symptom relief directly after elective ITB revision. Following our troubleshooting protocol, dysfunction of the ITB system was confirmed. The cause of ITB dysfunction with CSF leakage around the pump appeared to be a curled up silicone layer surrounding the sutureless pump connector. As far as we know, this complication has not been described before.

As in this case, diagnostics in ITB pump system malfunction can be challenging. The absence of anomalies in conventional radiography, CSF aspiration via the side port, or CT with contrast injection does not preclude ITB system malfunction. If these studies are inconclusive and clinical suspicion of system failure is high, an Indium (In111 DTPA) SPECT-CT study should be obtained [4, 12], whereas an Indium (In111 DTPA) SPECT-CT study slowly infuses the radioisotope and mimics baclofen diffusion, thereby identifying, localizing, and visualizing malfunction with an increased sensitivity and specificity compared to other diagnostics [3, 6].

As described in this case report, the silicone layer surrounding the sutureless pump connector ensuring watertight connection between the pump and catheter was shown to be curled up, demonstrating a unique cause of ITB system dysfunction. Although the cause of malconnection remains unknown, it is conceivable that this occurred before or during connection of the sutureless pump connector to the pump at the time of elective revision in March 2022. Considering this, it is expedient for surgeons to inspect and verify the watertight connection of the sutureless pump connector prior to definite connection.

## Conclusion

Complications after ITB surgery are common and multifarious. However, to the best of our knowledge, malconnection as a result of a curled up silicone layer surrounding the sutureless pump connector has not been reported before. Therefore, surgeons must be aware of this potential complication and inspection of the watertight connector prior to definite connection is advised.
